# Chronic lateral ankle instability increases the likelihood for surgery in athletes with os trigonum syndrome

**DOI:** 10.1007/s00167-018-5183-0

**Published:** 2018-10-01

**Authors:** P. D’Hooghe, K. Alkhelaifi, E. Almusa, M. Tabben, M. G. Wilson, J. F. Kaux

**Affiliations:** Aspetar Qatar Orthopaedic and Sports Medicine Hospital Doha, Near Khalifa Stadium, Doha, Qatar

**Keywords:** Ankle, Posterior impingement, Lateral ligament injury, Ankle sprain, Os trigonum syndrome, Professional athlete

## Abstract

**Purpose:**

The etiology and incidence of os trigonum syndrome in professional athletes is highly variable. There is a paucity of data to ascertain why some athletes evolve towards surgery whilst others remain asymptomatic. We hypothesized that a lateral ligament ankle injury would increase the likelihood for surgery in those athletes with os trigonum syndrome.

**Methods:**

Eighty professional athletes with clinical and radiological signs of os trigonum syndrome were identified to ascertain the incidence of injury to the lateral ligamentous ankle complex (acute and chronic) by magnetic resonance imaging (MRI). This cohort was subdivided into 2 groups; a surgical (*n* = 40) and a non-surgical (*n* = 40) cohort. Surgical division was decided if (1) the clinical hyper-plantar flexion test was positive, (2) a positive diagnostic ultrasound-guided injection and (3) no improvement was observed after 6 weeks of conservative rehabilitation.

**Results:**

From the surgical cohort, 37 players (94.1%) had a chronic lateral ankle ligament injury on MRI, whilst 3 players (5.9%) had an acute lateral ankle ligament injury. Binary logistic linear modelling revealed that having a chronic lateral ligament injury increases the likelihood of os trigonum syndrome surgery by ten times compared to those with an acute lateral ligament injury.

**Conclusion:**

Professional athletes with chronic lateral ligament ankle injury have an approximate ten times greater risk for os trigonum syndrome surgery compared to athletes with acute lateral ligament ankle injury.

**Level of evidence:**

IV.

## Introduction

Os trigonum ankle syndrome refers to a posterior ankle impingement pathology, often characterised by posterior ankle pain in plantar flexion. It is frequently observed in athletes where the mechanism of injury is either overuse or direct trauma [15, 18, 21]. The clinical prognosis appears to be better in those presenting with overuse injuries rather than trauma [12, 19]. The incidence of os trigonum syndrome in the athletic population is variable, ranging between 1.7 and 50%. Available data also suggests that between 33 and 50% of athletes present bilaterally. There does not appear to be an increased prevalence between men and women, nor between different age groups [15]. Athletes who participate in dynamic agility sports such as football, require a high degree of plantarflexion strain, and are thus more likely to symptomatic if having os trigonum syndrome [12, 15, 18, 19, 21].

The etiology of os trigonum ankle syndrome in professional athletes is highly variable. Although blunt trauma is considered a primary etiological factor, there is a paucity of data to why certain athletes evolve towards surgery whilst others remain asymptomatic for this condition. Most commonly, symptomatic os trigonum may be attributed to repetitive microtrauma due to impingement of the ossicle between the calcaneus and the postero-inferior aspect of the tibia [12, 15, 18, 19, 21]. Understanding why some athletes evolve towards surgery, and others not, have significant implications for those athletes with os trigonum syndrome regarding strategies for prevention, diagnosis, therapy and rehabilitation.

Diagnosing os trigonum syndrome is accomplished via a number of procedures. First, a clinical hyperplantar flexion test is considered positive for posterior impingement if it causes posterior apprehensive ankle pain. Secondly, a diagnostic ultrasound-guided injection may prove helpful in identifying the exact location of the pain [21]. Finally, radiological imaging often reveals a bony cause typical of os trigonum, that, if restricting athletic participation through impingement pain, is a clear indication for arthroscopic surgical resection [1, 4, 8, 15, 18, 21]. Arthroscopic treatment of posterior impingement provides excellent results and clinical improvement in the athlete’s ankle [9] with an expected post-operative return to training from 5 weeks onwards [3, 22].

Accordingly, the aim of the study was to ascertain if chronic lateral ankle instability is a contributing factor that leads an athlete with os trigonum syndrome towards surgery. Our hypothesis was that lateral ligament injury to an athlete’s ankle can increase the likelihood for surgery in those with os trigonum syndrome.

## Materials and methods

The pre-operative magnetic resonance images (MRI) of 80 professional athletes who were referred for surgical consultation at the Aspetar Hospital Orthopaedic Surgery Department during the past 5 years (2013–2017), were evaluated for the presence of chronic or acute lateral ligament complex injury. Inclusion criteria were the confirmation of an os trigonum on MRI imaging, together with having a positive hyper-plantar flexion clinical test and being a registered professional athlete with the Qatar Olympic Committee. Exclusion criterium was having previous ankle surgery or a malleolar fracture (lateral or medial).

A radiologist blinded to the research question at Aspetar Hospital was asked to review all ankle MRI images. The radiologist was asked to answer the following five radiological questions for each athlete [22]:

1. Confirmation of os trigonum in the ankle: yes/no.
2. Anterior talo-fibular ligament status: grade 1–3.
3. Calcaneo-fibular ligament status: grade 1–3.
4. Posterior talo-fibular ligament status: grade 1–3.
5. Acute (< 6 weeks after trauma) or chronic ligament injury (≥ 3 months after trauma) along the current radiological MRI guidelines [22].

This cohort was subdivided into two groups; a surgical (*n* = 40) and a non-surgical (*n* = 40) cohort. The division was decided if clinical hyper-planter flexion test was positive, if the ultrasound-guided injection was positive, and if no improvement was observed after 6 weeks of conservative rehabilitation.

All athletes were clinically evaluated by the same orthopaedic ankle expert and all were diagnosed with a positive clinical posterior ankle impingement test and a positive os trigonum finding on MRI. This study received IRB approval (#E2017000260) from the Qatar Anti-Doping Laboratory IRB.

### Statistical analysis

Data were analyzed using SPSS software (IBM-SPSS statistics, v23, Chicago, Illinois). Data were divided into two groups: surgical vs. non-surgical. Data are presented as count (percentage) of chronic vs. acute lateral ankle ligament injury in each group. No calculation of sample size was performed as this is a case–control study in which we included all possible cases from 2013 to 2017. A binary logistic linear model was used to analyze the association between the lateral ankle ligament injury (acute vs. chronic) and surgery outcome. Regression coefficients are presented as odds ratios with 95% confidence intervals (CI). *P* values < 0.05 were considered as statistically significant.

## Results

All forty professional athletes that required os trigonum surgery had some involvement of lateral ligament injury to the ankle on MRI; 37 players (94.1%) had a chronic lateral ligament injury [Table 1]. Three (5.9%) athletes that underwent os trigonum surgery, had an acute lateral ankle ligament injury on MRI.

**Table 1:** Surgery outcome vs. lateral ligament of the ankle (acute/chronic) cross-tabulation

	Ligaments state		Total
	Count of chronic case	Count of acute case	
Surgery			
No	28 (60.9%)	18 (39.1%)	46
Yes	32 (94.1%)	2 (5.9%)	34

Binary logistic linear model revealed that professional athletes with os trigonum syndrome were 10-times more likely to require surgery if presenting with a chronic lateral ligament injury of ankle for compared to those athletes with an acute lateral ligament injury (Table 2).

**Table 2:** Lateral ligament of the ankle (acute / chronic) risk factor for surgery in athletes with os trigonum syndrome

	Odds ratio	95% CIs	*P* value
Lateral ligament of the ankle			
Acute	1		
Chronic	10.3	2.2–48.3	0.003

Regression coefficients are presented as odds ratio with 95% CI

## Discussion

The most important finding of this study was that athletes presenting with chronic lateral ankle instability and os trigonum syndrome were ten times more likely to undergo surgery than athletes with an acute ankle injury and os trigonum syndrome. Although overuse and repetitive trauma (contact, plantar flexion and supination) are considered primary etiological factors for os trigonum syndrome, there is a paucity of data as to why certain athletes evolve towards surgery whilst others remain asymptomatic with this condition [5, 6, 16, 17].

The os trigonum syndrome mechanism of injury has been described as a “nut in a nutcracker” because the posterior talus and surrounding soft tissues are compressed between the tibia and the calcaneus during plantar flexion of the foot [2, 17]. Due to the repetitive plantar flexion movements in dynamic sports such as football, the chronic stress imposed on the posterior ankle increases the risk of developing osseous and soft-tissue injuries [2, 6, 17].

The two bony structures that are involved in the os trigonum syndrome impingement mechanism are the posterior tibial malleolus (which may have a prominent downward slope) and the superior surface of the calcaneal tuberosity (which may have a prominence) [13, 14]. The soft-tissue components of this anatomic interval, include the synovial sheath of the Flexor Hallucis Longus (FHL) and the posterior synovial recess of the tibiotalar and subtalar joints, all of which may be involved in this impingement syndrome. In fact, the reported prevalence of FHL tenosynovitis is greater in athletes with posterior ankle pain [6, 13, 16].

The differential diagnosis of os trigonum syndrome is considerable, with lateral ankle instability considered one of many conditions accounting for hindfoot ankle pain [2]. There are, however, reports suggesting that lateral ankle instability might be linked to mechanisms leading towards posterior ankle impingement [17]. In those athletes with a deficient lateral ligament complex as a result of an ankle inversion sprain for example, the talus can rotate more anteriorly under the tibial plafond. This can lead to an increased osseous impingement between the posterior tibia and the talus, resulting in os trigonum syndrome [13, 14]. A number of studies have demonstrated the link between lateral ankle instability and anterior impingement, but the link with posterior impingement has not been well documented [3, 10, 17, 21, 22]. Consequently, the difference between acute vs. chronic lateral instability and posterior impingement has never been fully proven.

**Fig. 1:**
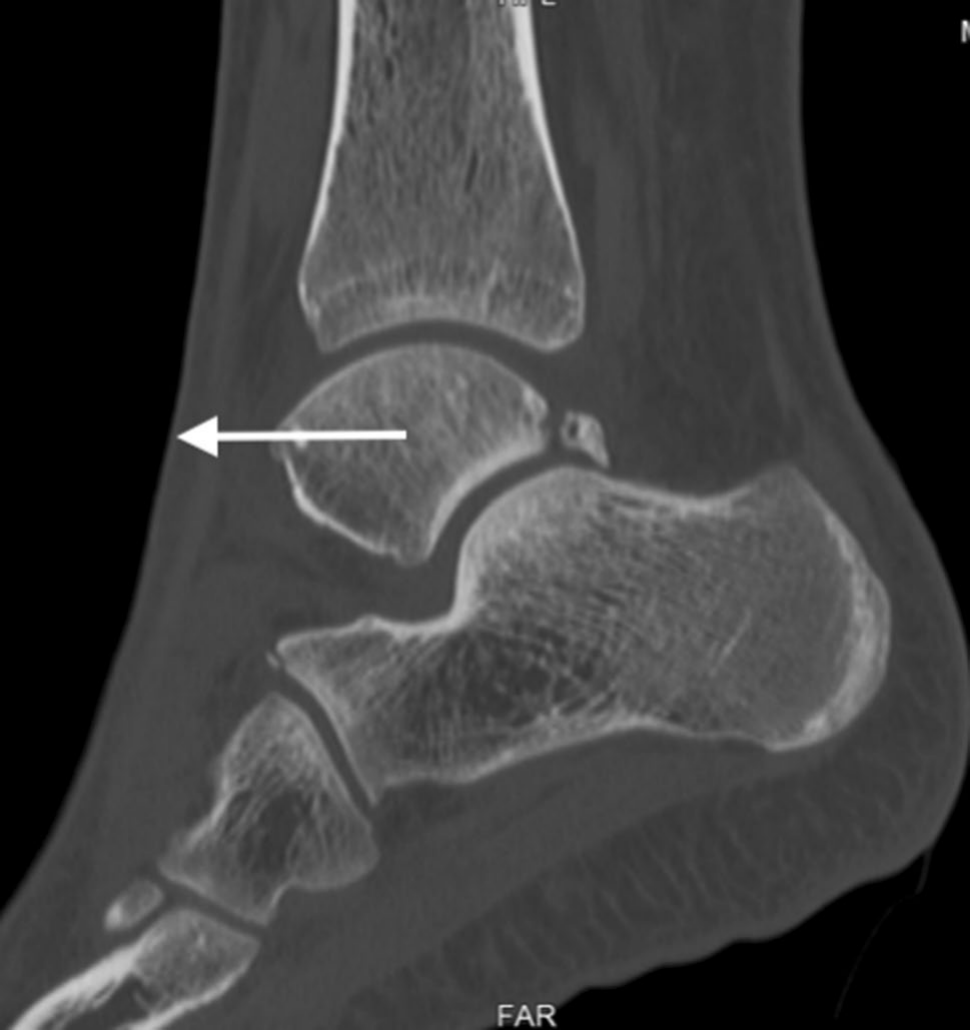
Anterior translation of the ankle (white arrow) in an athlete with os trigonum syndrome and combined chronic lateral ligament instability of the ankle

Biomechanical testing has demonstrated that a severe ankle sprain creates a pathological anterior translation of the ankle (Fig. 1), but not a posterior translation [7]. The implication of this means that the hyper plantar-flexion position of the ankle could create a greater mechanical posterior ankle conflict in the presence of a combined lateral ankle instability. These two important findings could be the reason why chronic lateral ligament instability is a key variable in the development of os trigonum syndrome. It might also explain why bony anterior impingement is frequently observed in combination with a restricted ankle range of motion where this is rarely the case in bony posterior impingement.

**Fig. 2:**
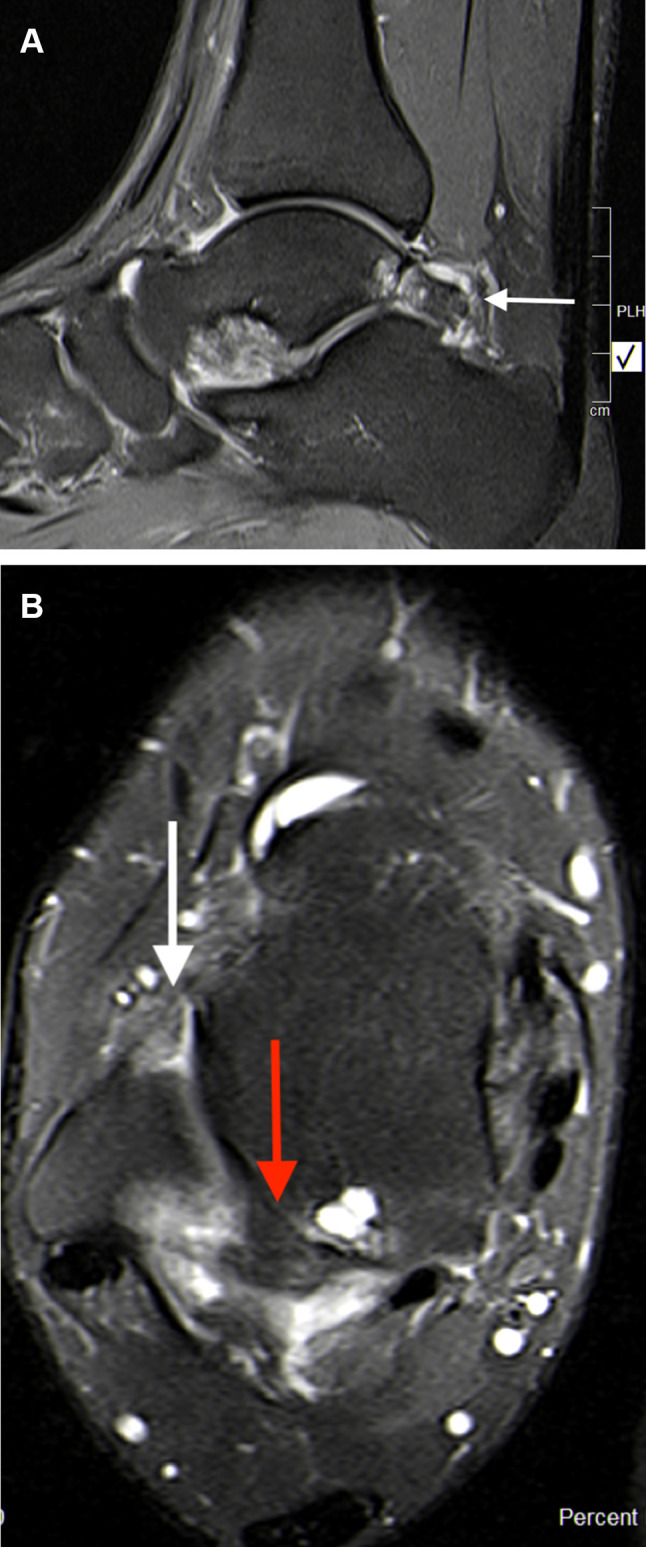
**a** Sagittal T2 MRI image depicting inflammatory signs and bony oedema over the os trigonum complex (white arrow). **b** Axial T2 MRI image depicting inflammatory signs and bony oedema over the os trigonum (red arrow) in an athlete with a combined chronic injury to the lateral ligament complex (white arrow)

An MRI study in athletes with os trigonum syndrome (sagittal T1-weighted and fat-suppressed T2-weighted images) observed abnormal signal intensity in the lateral talar tubercle and/or os trigonum, consistent with bone marrow edema [11]. Considering the mechanisms of injury, this abnormal signal intensity could be the result of bone impaction which represents micro-trabecular fractures, edema, and/or hemorrhage of the bone marrow without disruption of the cortex. Most ankle sprains occur in plantar flexion and inversion [10, 20]. When considering the repetitive dynamic movements involved in agility sports, this ankle position in those athletes with a deficient lateral ligament might explain why the os trigonum undergoes increased mechanical overload, becoming painful compared to incidental findings of os trigonum. This is supported with MRI findings of bone marrow edema over the os trigonum after hyper plantar flexion injury of the ankle (Fig. 2a, b).

In the present study, chronicity of ankle instability correlates with a significantly greater likelihood of os trigonum surgery compared to athletes with acute instability. We suspect that the initial swelling and overall dysfunction after acute ankle sprain limits the hyper plantar-flexion position of the ankle, and therefore, restricts the chance of posterior conflict. In addition to that, the ankle may also still count on its neuromuscular compensatory mechanisms in an acute ankle sprain.

This study is limited by its respective cross-sectional design. Further, no follow-up MRI scans were performed to ascertain the post-operative evolution of MRI signal intensity over time. Finally, no sample size calculation was undertaken and no test–retest reliability (clinical examination and MRI interpretation) was measured.

The clinical relevance of this study is that athletes with os trigonum syndrome should be investigated for combined chronic lateral ligament instability. By preventing ankle injuries in athletes with os trigonum evolving towards chronic lateral ankle instability, the likelihood of surgery might be significantly reduced.

To our knowledge, no previous studies have examined the association between the injury to the lateral ligament complex of the ankle (acute and chronic) and the clinical os trigonum syndrome. Consequently, this study offers new insights into the etiology and pathophysiology of posterior impingement in the athlete’s ankle. It also provides new evidence-based diagnostic indications for os trigonum syndrome surgery. More studies are necessary to evaluate the exact role of ankle instability in os trigonum syndrome, especially in the professional athletes and whether preventing an acute ankle injury from progressing into a chronic syndrome reduces the likelihood of surgery in those athletes with os trigonum syndrome.

## Conclusion

Professional athletes who have os trigonum syndrome and a chronic lateral ligament ankle injury have an approximate 10 times greater risk for surgery compared to athletes with os trigonum syndrome and an acute lateral ligament ankle injury.
